# Supplementary material for: Occurrence of “under-the-radar” antibiotic resistance in anthropogenically affected produce
Source: ISME J. 2025 Feb 6;19(1):wrae261. doi: 10.1093/ismejo/wrae261 (PMC11833317; doi:10.1093/ismejo/wrae261)
Supplement: Davidovitch_et_al_ISME_supplemental_material_submitted_wrae261 [file davidovitch_et_al_isme_supplemental_material_submitted_wrae261.pdf]

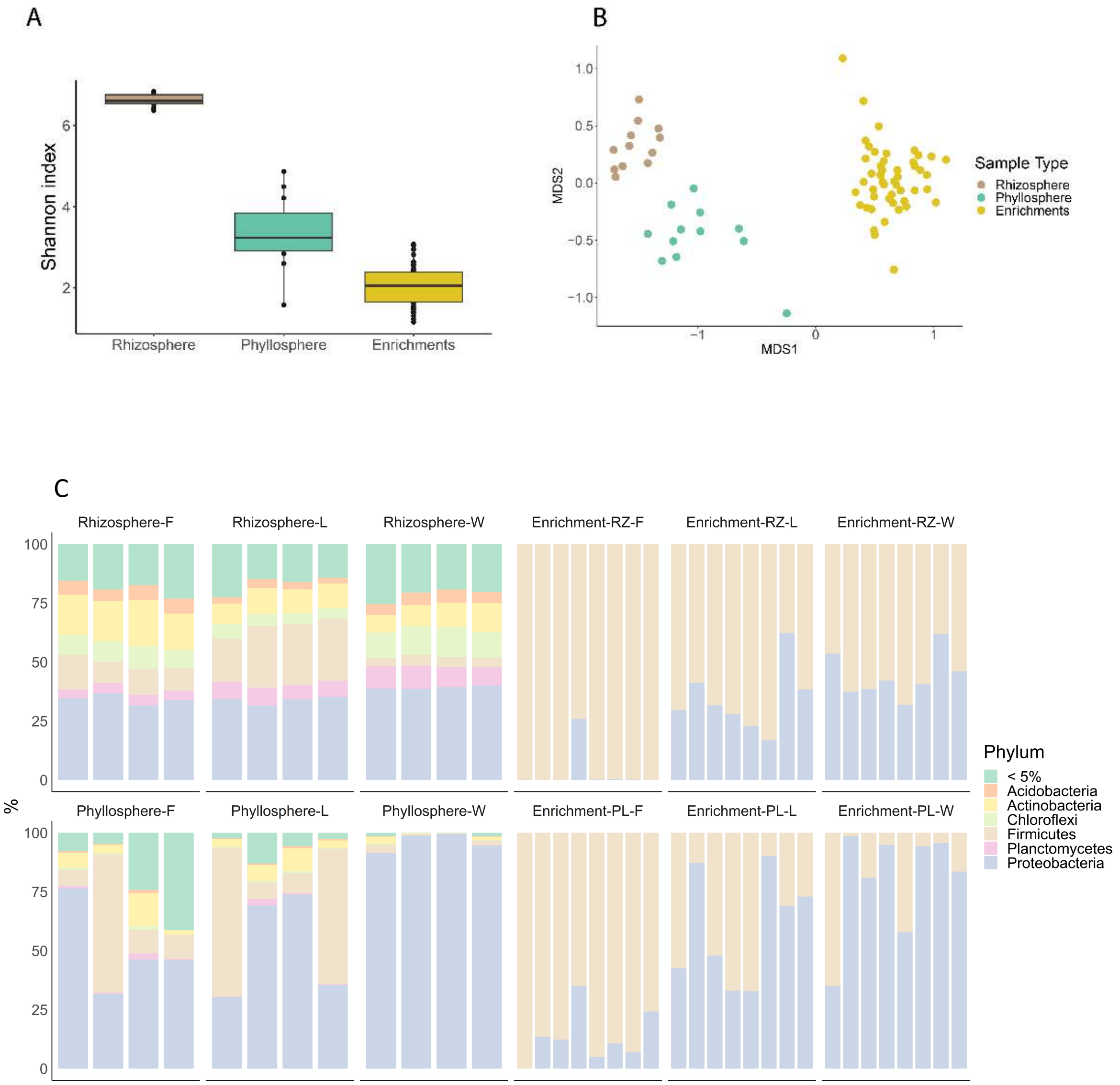

Supplementary Fig. 1. Impact of treatment and enrichment on bacterial diversity and community composition based on 16 rRNA gene amplicon sequencing data. **A** α-diversity based on the Shannon index of native and enriched samples (Wilcoxon test P value <0.00001. Figure 1A). **B** β-diversity of rhizosphere, phyllosphere, and enrichments bacterial communities using Bray–Curtis distance matrix and visualized by NMDS plot analysis (PERMANOVA, P value < 0.05) **C** Bacterial community composition (phylum-level) of source and enriched rhizosphere and phyllosphere samples. RZ= rhizosphere, PL= phyllosphere, F= freshwater, L= litter, W= TWW. <5% indicates families whose relative abundance represents < 5% of the total characterized bacterial community.

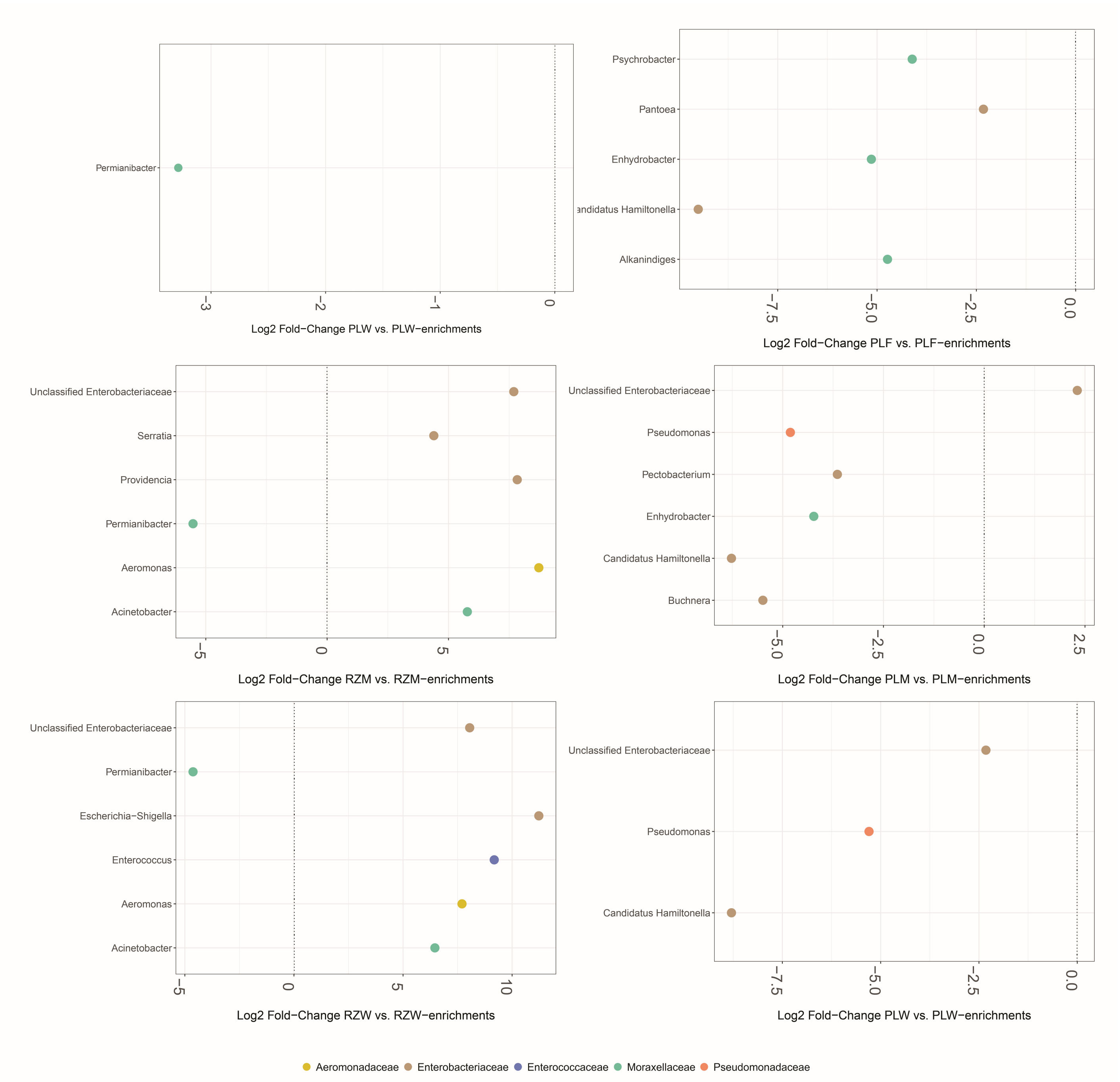

Supplementary Fig. 2. Differential abundance of genera from pathogen-associated families between enrichment and native samples. Positive log2 fold change values indicate higher abundance in enrichments, while negative values indicate higher abundance in native samples. (DESeq2 analysis: log2FoldChange > 1, adjusted *P* value < 0.01). RZ- rhizosphere; PL- phyllosphere. F- freshwater; L- litter; W-wastewater.

Factor	baseMean	log2FoldChange	lfcSE	stat	pvalue	padj	***Genus***
PLM	14344.84	6.035623	1.147809	-5.25839	1.45E-07	9.95E-06	*Bacillus*
PLM	27173.42	4.497438	1.401127	-3.20987	0.001328	0.006738	*UC Enterobacteriaceae*
PLW	1100.901	3.095895	0.991174	-3.12346	0.001787	0.008749	*Bacillus*
RZM	229.7507	26.35921	3.091026	-8.52766	1.49E-17	1.18E-16	*Providencia*
RZM	2446.945	10.7966	2.665583	-4.05037	5.11E-05	9.94E-05	*Aeromonas*
RZM	12443.98	9.910753	1.384442	-7.15866	8.15E-13	3.29E-12	*UC Enterobacteriaceae*
RZM	1715.905	8.079696	1.585899	-5.09471	3.49E-07	8.1E-07	*Acinetobacter*
RZM	9.974079	7.60409	2.461528	-3.08917	0.002007	0.003345	*Serratia*
RZM	671.0401	7.070627	1.413969	-5.00055	5.72E-07	1.31E-06	*Clostridium sensu stricto 1*
RZM	32146.54	5.130042	0.561444	-9.13724	6.41E-20	7.15E-19	*Bacillus*
RZM	1056.85	2.304	0.701803	-3.28297	0.001027	0.001755	*Lysinibacillus*
RZW	1688.045	28.18871	3.078817	-9.1557	5.4E-20	3.44E-19	*Paraclostridium*
RZW	1521.663	13.8925	1.143573	-12.1483	5.86E-34	1.49E-32	*Escherichia-Shigella*
RZW	300.1966	11.55189	1.167782	-9.89216	4.5E-23	4.19E-22	*Enterococcus*
RZW	2913.691	8.253645	0.786566	-10.4933	9.28E-26	1.13E-24	*UC Enterobacteriaceae*
RZW	4155.529	7.841534	0.720064	-10.8901	1.29E-27	1.84E-26	*Aeromonas*
RZW	360.2252	6.724341	2.187663	-3.07376	0.002114	0.003321	*Acinetobacter*
RZW	8016.005	5.51849	0.881317	-6.26164	3.81E-10	9.62E-10	*Bacillus*
RZW	2794.81	4.703947	0.829026	-5.67407	1.39E-08	3.19E-08	*Clostridium sensu stricto 1*
Anoxic	892.8958	-7.63186	0.874408	8.728027	2.59E-18	3.81E-15	*Paraclostridium*
Anoxic	45.45833	-6.49051	0.7619	8.518847	1.61E-17	1.18E-14	*Terrisporobacter*
Oxic	3052.625	5.759358	0.856405	-6.72504	1.76E-11	8.60E-09	*Acinetobacter*
Oxic	150.6875	3.72663	0.713726	-5.22138	1.78E-07	6.52E-05	*Pseudomonas*
Anoxic	4.729167	-3.08037	0.629359	4.894462	9.86E-07	0.00029	*Morganella*
Oxic	4.0625	2.832888	0.623446	-4.54392	5.52E-06	0.001352	*Providencia*
Enrichments	3508.503	6.737762	1.43169	-4.70616	2.52E-06	1.04E-05	*Lactococcus*
Enrichments	9710.694	3.227303	0.747125	-4.31963	1.56E-05	5.48E-05	*Bacillus*

Supplementary table 1. Influence of enrichment, oxidation levels, and treatment factors on bacterial genera abundance. RZ- rhizosphere; PL- phyllosphere; L-litter; W- wastewater; UC-

unclassified (Deseq2 adjusted *P* value <0.0, Shown are genera with log2FoldChange > 2 or < -2).

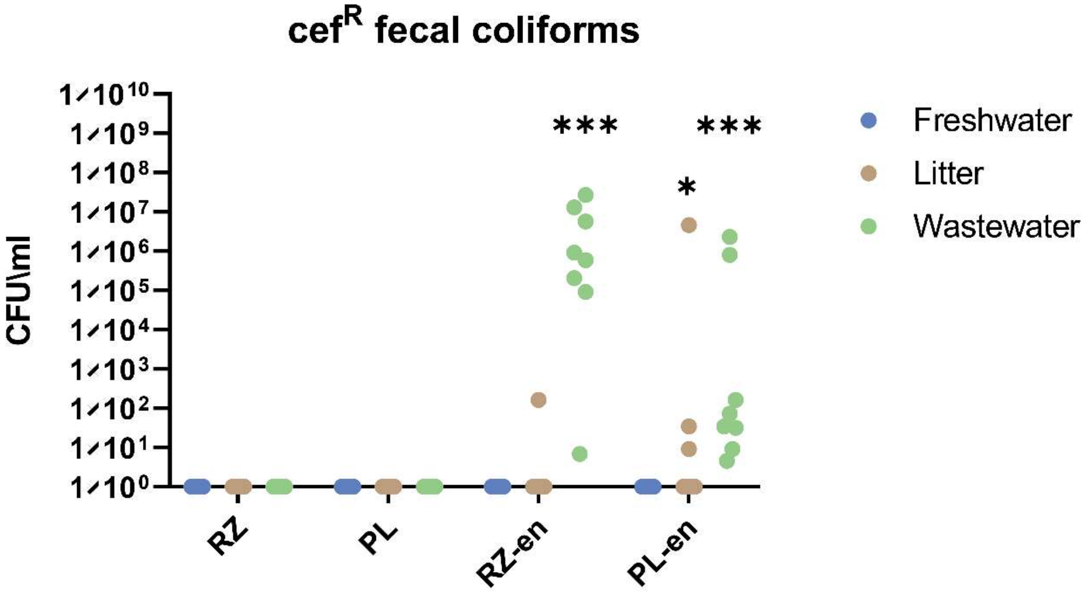

Supplementary Fig. 3. CFU/ml of cefotaxime resistant of total cefotaxime resistant fecal coliforms (Wilcoxon pairwise test *** *P* value <0.0001; * *P* value<0.01). RZ- rhizosphere; PL- phyllosphere; en-enrichments.

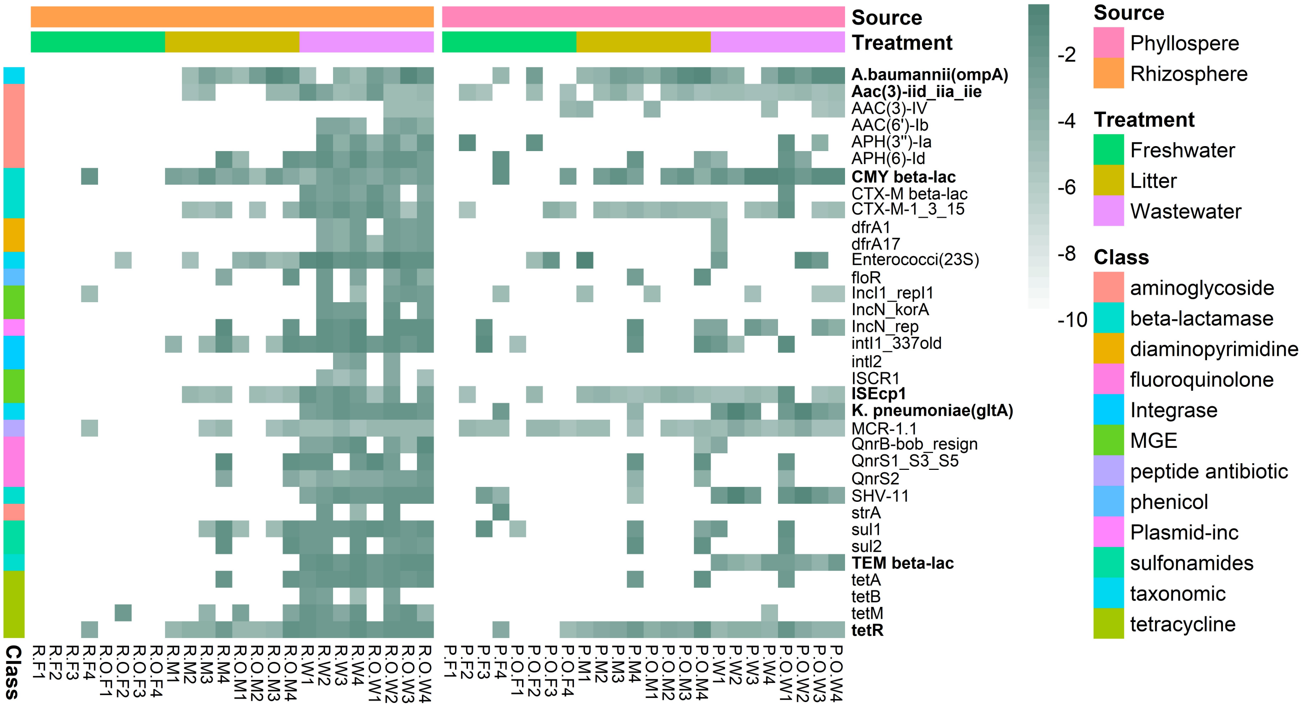

Supplementary Fig. 4. HT-qPCR analysis of enriched rhizosphere and phyllosphere samples targeting clinically relevant ARGs and taxa, showing gene markers with statistically significant differences between the three treatments. Bolded markers indicate that they were significant in both rhizosphere and phyllosphere anthropogenically associated enrichments (Wilcoxon Test *P* value <0.05)

A B

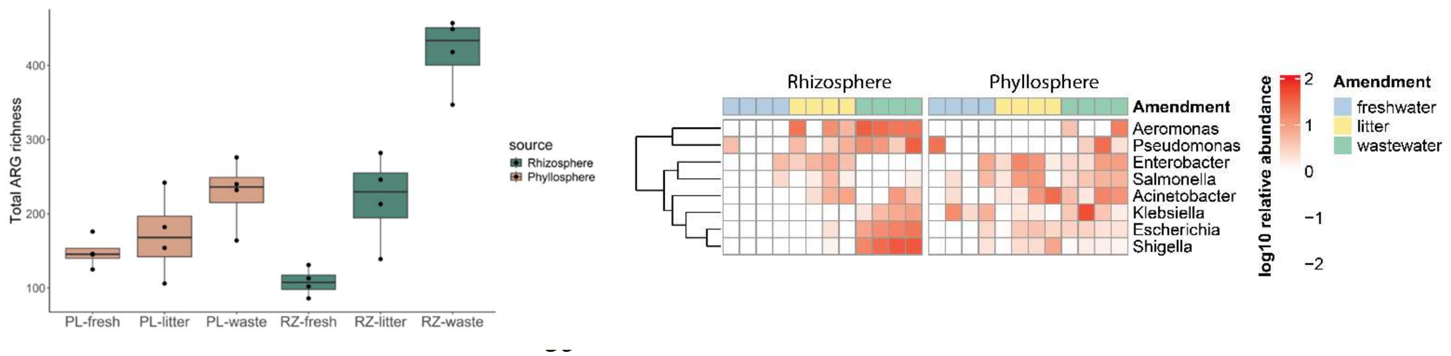

Supplementary Fig. 5. ARGs and taxa abundance and diversity based on metagenomic data. **A** ARGs richness of freshwater-irrigated, litter-amended, and TWW-irrigated enriched rhizosphere and phyllosphere samples (Wilcoxon test *P* value <0.05). **B** log10 relative abundance of clinically relevant genera in enriched samples (Deseq2 adjusted *P* value <0.01). RZ- rhizosphere; PL- phyllosphere; fresh- freshwater; waste- wastewater.

Isolate	Origin	Oxidation	Detected species	Score
C-PLO-M4-1	Litter-phyllosphere	Oxic	*K.pneumoniae*	2.32
C-PLO-M3-5	Litter-phyllosphere	Oxic	*K.pneumoniae*	2.35
C-PLO-M1-1	Litter-phyllosphere	Oxic	*K.pneumoniae*	2.04
C-RZO-M4-4	Litter-rhizosphere	Oxic	*K.pneumoniae*	2.16
C-PL-W4-4	TWW-phyllosphere	Anoxic	*E. kobei*	2.20
C-RZ-W4-5	TWW- rhizosphere	Anoxic	*K.pneumoniae*	2.37
C-PLO-M4-2	Litter-phyllosphere	Oxic	*E. asburiae*	2.17
C-PLO-M4-5	Litter-phyllosphere	Oxic	*A. baumannii*	2.14
C-PLO-M4-3	Litter-phyllosphere	Oxic	*E. kobei*	2.24

Supplementary table 2. MALDI results of isolates isolated from anthropogenically associated enrichments.

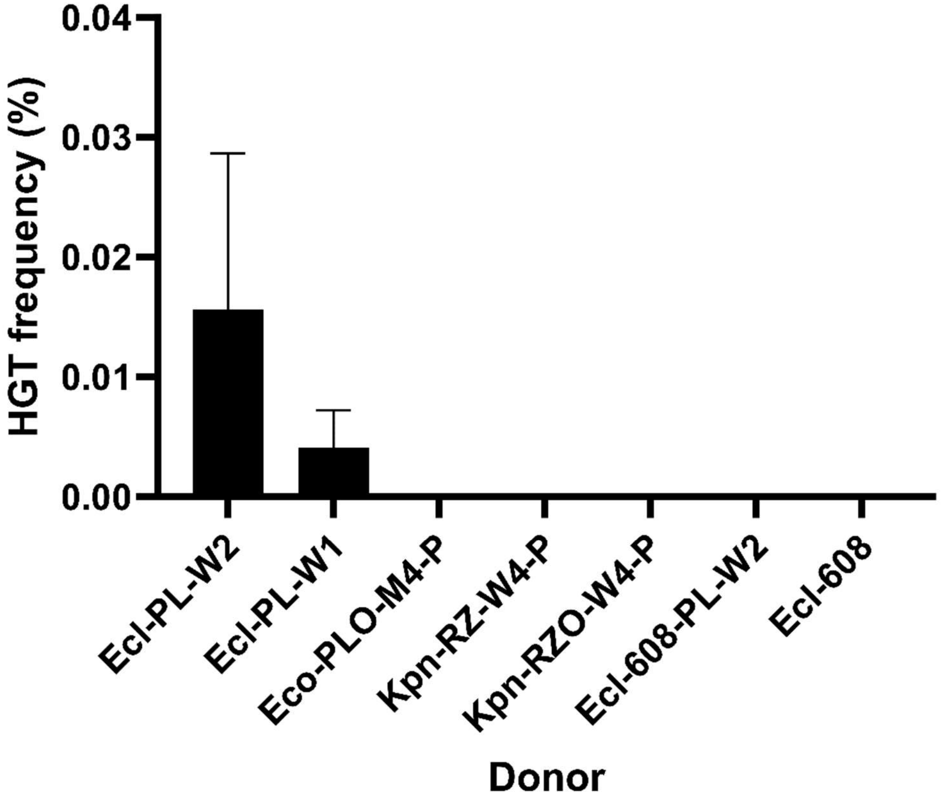

Supplementary Fig. 6. HGT of *E.coli 608* by fecal coliform strains isolated from litter- and TWW- associated enrichments represented as the percentage of transconjugants CFU/ml divided by recipients CFU/ml. Ecl- *E.coli*, Eco- *Enterobacter Cloacae*, Kpn- *Klebsiella pneumoniae*.
